# Fine-scale Identification of the Most Likely Source of a Human Plague Infection

**DOI:** 10.3201/eid1510.090188

**Published:** 2009-10

**Authors:** Rebecca E. Colman, Amy J. Vogler, Jennifer L. Lowell, Kenneth L. Gage, Christina Morway, Pamela J. Reynolds, Paul Ettestad, Paul Keim, Michael Y. Kosoy, David M. Wagner

**Affiliations:** Northern Arizona University, Flagstaff, Arizona, USA (R.E. Colman, A.J. Vogler, P. Keim, D. Wagner); Centers for Disease Control and Prevention, Fort Collins, Colorado, USA (J.L. Lowell, K.L. Gage, C. Morway, M.Y. Kosoy); New Mexico Department of Health, Santa Fe, New Mexico, USA (P.J. Reynolds, P. Ettestad)

**Keywords:** Molecular epidemiology, plague, *Yersinia pestis*, VNTR, MLVA, probabilistic modeling, bacteria, dispatch

## Abstract

We describe an analytic approach to provide fine-scale discrimination among multiple infection source hypotheses. This approach uses mutation-rate data for rapidly evolving multiple locus variable-number tandem repeat loci in probabilistic models to identify the most likely source. We illustrate the utility of this approach using data from a North American human plague investigation.

Linking human disease events to likely sources of infection has been advanced by molecular epidemiology. However, isolates from several potential infection sources often are similar, and none may exactly match the clinical isolate genotype, especially if the methods used provide high discrimination (*1*). Conclusions from partial-match genotypes are problematic but may provide the only data for weighing the relative importance of similar source genotypes. Even perfect-match genotypes do not preclude partial-match sources as likely infection sources (*2*). We present a probabilistic approach based on mutation rates that can be used to identify the most likely source of infection. Our example is human plague, but the approach could be applied to other diseases for which data on marker mutation rates are available (*3*).

Plague is caused by the bacterium *Yersinia pestis.* Because *Y. pestis* is an obligate pathogen that continuously cycles between rodents and fleas, mutations are generated regularly and can be observed among even closely related isolates (*1*). Human contact with infected fleas or rodents can result in human plague (*4*). Plague is rare in the United States, with <20 cases in 2006 (*5*) but is of concern because of the potential use of *Y. pestis* as a biological weapon (*6*). Thus, the ability to link a human plague isolate to a likely source has implications for investigating both natural disease and bioterrorism events.

Multiple locus variable-number tandem repeat (VNTR) analysis (MLVA) is useful for molecular epidemiologic studies of *Y. pestis* because of its discrimination power (*1*,*7*,*8*). We previously used MLVA to genotype the human isolate described below and queried the resulting genotype against a database containing genotypes from hundreds of *Y. pestis* isolates (*9*). This statistical approach identified isolates that most closely matched the human isolate and confirmed its most likely coarse geographic origin (northern New Mexico). However, this set of near matches from the database query included isolates representing several different potential local infection sources, leaving the most likely fine-scale source unclear. The human and environmental isolates were indistinguishable with pulsed-field gel electrophoresis (PFGE); thus, the most likely fine-scale source could not be identified (*10*).

## The Study

In November 2002, while visiting New York, New York, USA, 2 persons from Santa Fe County, New Mexico, USA, became ill with fever and unilateral inguinal adenopathy; clinicians subsequently identified the illness as bubonic plague. Investigation by the New Mexico Department of Health and the Centers for Disease Control and Prevention indicated the patients were infected in New Mexico because *Y. pestis*–positive fleas were collected near the patients’ home (*10*). However, because plague is endemic to the region, and flea samples from which isolates were obtained were collected at the home and along a local trail on which the patients hiked, either location could be the source. To identify the most likely fine-scale source of their infections, we examined specific mutations separating the human isolate from closely related environmental isolates.

**Figure 1:**
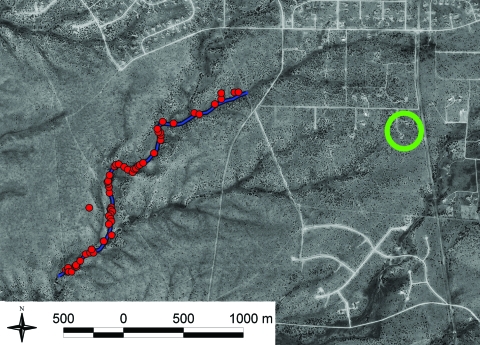
Distribution of rodent trapping stations along a hiking trail in Santa Fe County, New Mexico, USA. Each red circle indicates a single trapping site that had 3 traps. Trap stations (not shown) also were placed throughout the patients’ yard (green circle).

We examined 5 *Y. pestis* isolates (Table 1) to develop a fine-scale spatial analysis of the infection. The reference isolate was obtained from 1 patient, 3 isolates were obtained from fleas collected in the patients’ yard (*9*) (2 were collected before their illness as part of a long-term investigation), and 1 isolate was obtained from the trail flea samples a short time later as part of the same long-term study (Figure 1). Other isolates were collected and examined but were excluded from this fine-scale analysis because they were more distinct from the human isolate, differing at >4 VNTR loci. DNA extracts were prepared from each isolate (*11*,*12*) and analyzed using a 43-loci MLVA system as previously described (*1*,*8*).

**Table 1:** Five *Yersinia pestis* isolates examined to determine the source of a human plague infection in New Mexico, USA*

CDC isolate ID	Collection date	Collection source	MLVA genotype†	Flea source of *Y. pestis* isolate	Rodent source of flea
NM024452	2002 Nov 5	Human	A	NA (human)	NA (human)
NM02-1852-138	2002 Jul 17	Yard	B	*Orchopeas sexdentatus*	*Neotoma micropus*
NM02-1856-140	2002 Jul 18	Yard	B	*O. neotomae*	*N. micropus*
NM02-4477-309	2002 Nov 9	Yard	B	*Peromyscopsylla hesperomys*	*Peromyscus leucopus*
ED425	2003 Apr 4	Trail	C	*O. sexdentatus*	*N. micropus*

**Figure 2:**
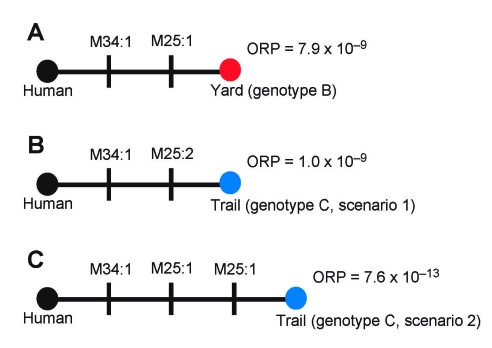
Alternate infection source hypotheses for the plague cases in the persons who visited New York, New York, USA. Closed circles indicate genotypes; black, red, and blue circles indicate genotypes A, B, and C, respectively. Individual mutations are indicated as vertical lines on the comparisons and are labeled with the locus that mutated and the number of repeats involved in the mutations. Overall relative probabilities (ORP) based on *Yersinia pestis* mutation rates are presented for each comparison.

*CDC, Centers for Disease Control and Prevention; ID, identification number; MLVA, multiple locus variable-number tandem repeat analysis; NA, not applicable. †See Figure 2.

We observed 3 MLVA genotypes (A–C) among the 5 samples (Table 1, Figure 2). The human isolate was assigned genotype A. Genotype B, observed in 3 isolates obtained from the yard, differed from the reference by single-repeat mutations at 2 VNTR loci (M25 and M34; Figure 2, panel A). Genotype C, observed in 1 isolate from a flea obtained along the trail, also differed from the reference isolate at loci M25 and M34. However, the mutation at M25 was a double-repeat mutation that could be explained 2 ways: as a single 2-repeat mutational event (Figure 2, panel B) or as 2 sequential single-repeat mutations at the same locus (Figure 2, panel C). Although all 43 VNTR loci are useful for identifying the coarse geographic origin of an unknown isolate by using a database approach (*9*), our analysis examined only polymorphic loci because monomorphic loci provided no additional information. The molecular epidemiologic goal was to identify the environmental isolate most closely related to the human isolate and thus the most likely fine-scale geographic source of the infection.

To this end, we examined the relative probability of each mutation (Table 2) using published mutation rate data (*1*,*8*). We used mutation rate estimates for specific mutational events to judge relative probabilities of different scenarios. This approach assumes 1) there is an intrinsic mutation rate at each loci for each event, 2) we have accurately estimated these rates (*1*,*8*), and 3) we can use intrinsic rates to judge the relative likelihood of >2 hypotheses. We multiplied individual probabilities of mutations within a scenario to calculate the overall relative probability (ORP) that an environmental isolate was related to the infection source (Table 2; Figure 2). To select the most likely source, we compared the ORP of each scenario with the others in a pairwise fashion (odds ratios, Table 2). In practice, only the most likely source needs to be compared with all other sources.

**Table 2:** Overall relative probabilities of isolates with genotypes B or C as the source of a human plague infection in New Mexico, USA*

MLVA genotype	Rates of specific mutations between each genotype and genotype of the human isolate (A)†			Hypothesis	Overall relative probability	OR‡
	M34:1	M25:1	M25:2			
B (yard)	8.2 × 10^–5^	9.7 × 10^–5^	–	B→A	7.9 × 10^–9^	–
C (trail, scenario 1)	8.2 × 10^–5^	–	1.3 × 10^–5^	C1→A	1.0 × 10^–9^	7.9
C (trail, scenario 2)	8.2 × 10^–5^	(9.7 × 10^–5^)^2^	–	C2→A	7.6 × 10^–13^	1.0 × 10^4^

*MLVA, multiple locus variable-number tandem repeat analysis; OR, odds ratio. †Values generated using data and approaches described in (*8*). ‡The overall relative probability for each subsequent hypothesis is compared with the most likely hypothesis (B→A).

## Conclusions

The patients most likely were infected from a source in their yard. Genotype B was observed in isolates from the yard, and this scenario had the highest ORP (7.9 × 10^–9^; hypothesis B→A; Table 2). The first scenario for genotype C (C1→A; Table 2) is second most likely (ORP 1.0 × 10^–9^). The odds ratio shows the most likely scenario (B→A) is just 7.9× more likely than this scenario (C1→A). These 2 near matches illustrate the power of this approach: one is the most likely source, but the other is statistically possible because this odds ratio difference would not be significant at α<0.05 (odds ratio >20). However, the ORP (1.0 × 10^4^) for the second scenario for genotype C (C2→A; Table 2) would be statistically significant, enabling it to be rejected.

When a high-resolution typing approach based on loci with fast mutation rates, such as MLVA, is used, near matches should be the rule rather than the exception. After transmission, the pathogen will continue to propagate in environmental sources and in the patient, leading to additional mutations before investigators obtain isolates. Mutations may also occur during routine laboratory procedures (e.g., culturing) before genotypic comparisons. Thus, perfect matches are rarely observed during phylogenetic analysis. Rather, the common ancestor (i.e., genotype of the source strain at time of infection) of the human isolate and each potential source isolate will need to be hypothesized. MLVA and probabilistic modeling provide a rigorous means to identify the most likely fine-scale environmental source. The same principles can be applied to other subtyping approaches used in investigations, including those with slower evolution patterns such as PFGE. In these cases, matches and near matches also should be judged by their relative evolutionary rates. Applying evolutionary probabilistic modeling to subtyping will generate stronger conclusions by evaluating the relative strengths of alternative hypotheses regardless of the subtyping approach.
